# Effects of Soluble Epoxide Hydrolase Deficiency on Acute Pancreatitis in Mice

**DOI:** 10.1371/journal.pone.0113019

**Published:** 2014-11-17

**Authors:** Ahmed Bettaieb, Samah Chahed, George Tabet, Jun Yang, Christophe Morisseau, Stephen Griffey, Bruce D. Hammock, Fawaz G. Haj

**Affiliations:** 1 Department of Nutrition, University of California Davis, Davis, California, United States of America; 2 Department of Entomology and Nematology, University of California Davis, Davis, California, United States of America; 3 Comprehensive Cancer Center, University of California Davis, Sacramento, California, United States of America; 4 Comparative Pathology Laboratory, University of California Davis, Davis, California, United States of America; 5 Division of Endocrinology, Diabetes and Metabolism, Department of Internal Medicine, University of California Davis, Sacramento, California, United States of America; University of Munich, Germany

## Abstract

**Background:**

Acute pancreatitis (AP) is a frequent gastrointestinal disorder that causes significant morbidity, and its incidence has been progressively increasing. AP starts as a local inflammation in the pancreas that often leads to systemic inflammatory response and complications. Soluble epoxide hydrolase (sEH) is a cytosolic enzyme whose inhibition in murine models has beneficial effects in inflammatory diseases, but its significance in AP remains unexplored.

**Methodology/Principal Findings:**

To investigate whether sEH may have a causal role in AP we utilized *Ephx2* knockout (KO) mice to determine the effects of sEH deficiency on cerulein- and arginine-induced AP. sEH expression increased at the protein and messenger RNA levels, as well as enzymatic activity in the early phase of cerulein- and arginine-induced AP in mice. In addition, amylase and lipase levels were lower in cerulein-treated *Ephx2* KO mice compared with controls. Moreover, pancreatic mRNA and serum concentrations of the inflammatory cytokines IL-1B and IL-6 were lower in cerulein-treated *Ephx2* KO mice compared with controls. Further, *Ephx2* KO mice exhibited decreased cerulein- and arginine-induced NF-κB inflammatory response, MAPKs activation and decreased cell death. *Conclusions* -These findings demonstrate a novel role for sEH in the progression of cerulein- and arginine-induced AP.

## Introduction

Acute pancreatitis (AP) is a frequent gastrointestinal disorder that causes significant morbidity [1], [2]. The incidence of AP has been progressively increasing in parallel with its risk factors such as obesity, alcohol abuse and duct obstruction by gallstones [2], [3]. The onset of the disease is thought to be triggered by intra-acinar cell activation of digestive enzymes such as trypsinogen that results in interstitial edema, inflammation and acinar cell death that often lead to systemic inflammatory response and complications [4]–[6]. Specific therapy for AP is lacking and deciphering the molecular mechanisms underlying its pathogenesis will likely aid in therapeutic intervention.

Several animal models have been utilized to study the pathogenesis of AP; one of the most common is cerulein-induced pancreatitis [7]. Cerulein is an ortholog of the intestinal hormone cholecystokinin and at high concentrations cause death of acinar cells and infiltration of inflammatory cells into the pancreas, which are also observed in human pancreatitis [8], [9]. The mechanism of cerulein action involves activation of NF-κB and the release of pro-inflammatory cytokines such as TNFA, IL-1B and IL-6 [10], [11]. TNFA and IL-1B are primary cytokines that initiate and propagate most the systemic inflammatory response [12], [13], while IL-6 mediates the acute-phase response [14]. Pro-inflammatory cytokines activate the IκB kinase complex (IKK) to phosphorylate inhibitor of NF-κB (IκB) [15]. IκB phosphorylation triggers its ubiquitination and subsequent degradation, leading to the dissociation of NF-κB dimers to the nucleus for activation of transcription [16].

Soluble epoxide hydrolase (sEH) is a ubiquitously expressed predominantly cytosolic enzyme with C-terminal epoxide hydrolase and N-terminal lipid phosphatase activities [17], [18]. Endogenous substrates for sEH include epoxy fatty acids such as epoxyeicosatrienoic acids (EETs) which are arachidonic acid metabolites produced by cytochrome P-450 epoxygenases. sEH plays an important role in regulating the level of EETs and other epoxide containing lipids by effectively degrading them into the less potent metabolites, dihydroxyeicostrienoic acids (DHETs) [19]. EETs are more biologically active than DHETs and the other corresponding fatty acid diols which are rapidly conjugated and excreted [20]. Insights into the physiological functions of sEH have emerged from studies in mice with global *Ephx2* deficiency and the development of selective sEH pharmacological inhibitors (sEHI) [21]. sEH pharmacological inhibition has beneficial effects in cardiovascular, renal, metabolic and inflammatory diseases in murine models [22]–[27]. sEH inhibition stabilizes EETs and other epoxy fatty acids by preventing their conversion to DHETs or the other corresponding fatty acid diols [28]. The stabilized EETs have anti-inflammatory effects through inhibition of NF-κB and IκB [29]. Further, sEHI can also synergize with conventional anti-inflammation drugs, e.g. non-steroidal anti-inflammatory drugs to reduce inflammation [30]. Importantly, sEH deficiency and prolonged pharmacological inhibition in mice appear to be quite benign [24]. Given the salutary effects of sEH deficiency, it is an attractive target for therapy of several chronic diseases.

The role of sEH in AP has heretofore remained unexplored but a growing body of evidence implicates sEH in pancreatic endocrine function. sEH deficiency and pharmacological inhibition promote insulin secretion and reduce islet apoptosis in a type 1 diabetes model [31], [32] and increase islet mass in a mouse model of high fat diet-induced insulin resistance [24]. While these findings provide insights into the function of sEH in pancreatic islets, its role in acinar cells remains largely unknown. In the present study, the effects of sEH deficiency on cerulein- and arginine-induced AP were investigated. Alterations in systemic inflammation were determined in cerulein- and arginine-treated versus non-treated control and *Ephx2* knockout (KO) mice, and the underlying molecular mechanism investigated.

## Methods

### Mouse studies

Mice with targeted disruption in exon 1 of the *Ephx2* gene [33], were back-crossed onto a C57BL/6J background (Jackson Laboratories) [24]. Mice were maintained on a 12 h light-dark cycle in a temperature-controlled facility, with free access to food and water. All studies were performed using male mice. *Ephx2* KO and wild type (WT) male mice were fed standard laboratory chow (Purina’s Lab Diet, #5001) at weaning. Acute pancreatitis was induced in 8–12 week old male WT and KO mice using cerulein or arginine. For cerulein-induced AP, mice were fasted overnight then injected intraperiotoneally with cerulein (50 µg/kg body weight) 12 consecutive times, at 1 h intervals. The control group was administered DMSO as a vehicle control for cerulein. Animals were sacrificed 2 h after the last injection (14 h after the initial injection of cerulein) and blood was collected to determine serum lipase and amylase using commercial kits (Sigma) according to the manufacturer’s instructions. Levels of serum cytokines were measured using a Multiplex kit (Meso Scale Discovery) according to the manufacturer’s protocol. Another group of animals was used for arginine-induced AP as previously described, with modifications [34]. Briefly, mice received a single intraperitoneal injection of 5 g/kg body weight L-arginine monohydrochloride in 0.9% sodium chloride (pH: 7.0). Animals were sacrificed 48 and 72 h after arginine injection. All mouse studies were conducted according to federal guidelines and approved by the Institutional Animal Care and Use Committee at University of California Davis.

### Biochemical studies

Pancreata were lysed using radio-immunoprecipitation assay (RIPA) buffer (10 mM Tris-HCl, pH: 7.4, 150 mM NaCl, 0.1% sodium dodecyl sulfate [SDS], 1% Triton X-100, 1% sodium deoxycholate, 5 mM EDTA, 1 mM NaF, 1 mM sodium orthovanadate and protease inhibitors). Lysates were clarified by centrifugation at 13,000 rpm for 10 min, and protein concentrations were determined using a bicinchoninic acid protein assay kit (Pierce Chemical) according to the manufacturer’s instructions. Proteins were resolved by SDS-PAGE and transferred to PVDF membranes. Immunoblotting of lysates and immunoprecipitates was performed with antibodies for sEH (generated by the Hammock laboratory), cleaved Caspases 8, 9 and 3, PARP, SHP1 and Tubulin (all from Santa Cruz), pp38 (Thr180/Tyr182), p38, pJNK (Thr183/Tyr185), JNK, pIKKα/β (Ser178/180), IKKα/β, pIκBα (Ser32), IκBα, pNF-κBp65 (Ser536), NF-κBp65 and NF-κBp50 (all from Cell Signaling). After incubation with the appropriate secondary antibodies, proteins were visualized using enhanced chemiluminescence (ECL, Amersham Biosciences). Pixel intensities of immunoreactive bands were quantified using ImageQuant 5.0 software (Molecular Dynamics). For phosphorylated proteins data are presented as phosphorylation level normalized to total protein expression (such as pIKKα^S178/180^/pIKKα) for each animal and for non-phosphorylated proteins as total protein expression normalized to Tubulin (such as sEH/Tubulin) for each animal.

Total RNA was extracted from pancreata using TRIzol reagent (Invitrogen). cDNA was generated using high-capacity cDNA synthesis Kit (Applied Biosystems). *Ephx2*, *Il1-β*, *Il-6* and *Tnfa* were assessed by SYBR Green quantitative real time PCR using SsoAdvanced Universal SYBR Green Supermix (iCycler, BioRad). Relative gene expression was quantitated using the ΔCT method with appropriate primers (Table 1) and normalized to *Tata-box binding protein (Tbp).* Briefly, the threshold cycle (Ct) was determined and relative gene expression was calculated as follows: fold change = 2-Δ(ΔCt), where ΔCt = Ct target gene-Ct TBP (cycle difference) and Δ(ΔCt) = Ct (treated mice)−/Ct (control mice).

**Table 1:** Primer sequences used to quantitate *sEH*, *Il-1b*, *Il-6*, *Tnfa* and *Tbp* expression.

Gene	Forward 5′->3′	Reverse 5′->3′
Il-1b	AGCTTCAGGCAGGCAGTATC	AAGGTCCACGGGAAAGACAC
Il-6	ACAACCACGGCCTTCCCTACTT	CACGATTTCCCAGAGAACATGTG
Ephx2	CTGGATACCCTGAAGGCAAA	TGACGTCATTTGGATTGCAT
Tbp	TTGGCTAGGTTTCTGCGGTC	GCCCTGAGCATAAGGTGGAA
Tnfa	GACGTGGAACTGGCAGAAGAG	TGCCACAAGCAGGAATGAGA

### Histological analyses

WT and *Ephx2* KO male mice were injected intraperiotoneally with cerulein or DMSO (50 µg/kg body weight) 12 consecutive times, at 1 h intervals then sacrificed 48 h after the first injection. A portion of the pancreas was fixed in 4% paraformaldehyde overnight, embedded in paraffin and 5 µm sections were stained with hematoxylin and eosin (H&E) to observe morphological changes. Histological analysis was initially performed in a blinded fashion. Histological scoring of pancreatic sections was performed to grade the extent of pancreatic parenchyma edema (0: no edema, 1: interlobular edema, 2: interlobular and moderate intralobular edema, 3: interlobular and severe intralobular edema), cell vacuolation (0: none, 1: <20% acini with vacuoles, 2: <50% acini, 3: >50% acini), inflammation (0: no inflammation, 1: inflammatory cells present at intralobular, 3: inflammatory cells present at interacini), and acinar cell necrosis (0: no necrosis, 1: <10% necrosis, 2: <40% necrosis, 3>40% necrosis) as previously described [35].

### Determination of levels of eicosanoids

Pancreata were homogenized as described in a previous publication [35] and extracted by solid phase extraction and reconstituted with internal standard solution. Then, samples were analyzed by reverse phase liquid chromatography tandem mass spectrometer (LC/MS/MS) under negative MRM mode [36].

### Statistical analyses

Data are expressed as means±standard error of the mean (SEM). Data comparisons were performed using Tukey’s-Kramer honest significant difference analyses using the JMP program (SAS Institute). Differences were considered significant at P≤0.05 and highly significant at P≤0.01.

## Results

### sEH expression is increased in the early phase of acute pancreatitis

**Figure 1:**
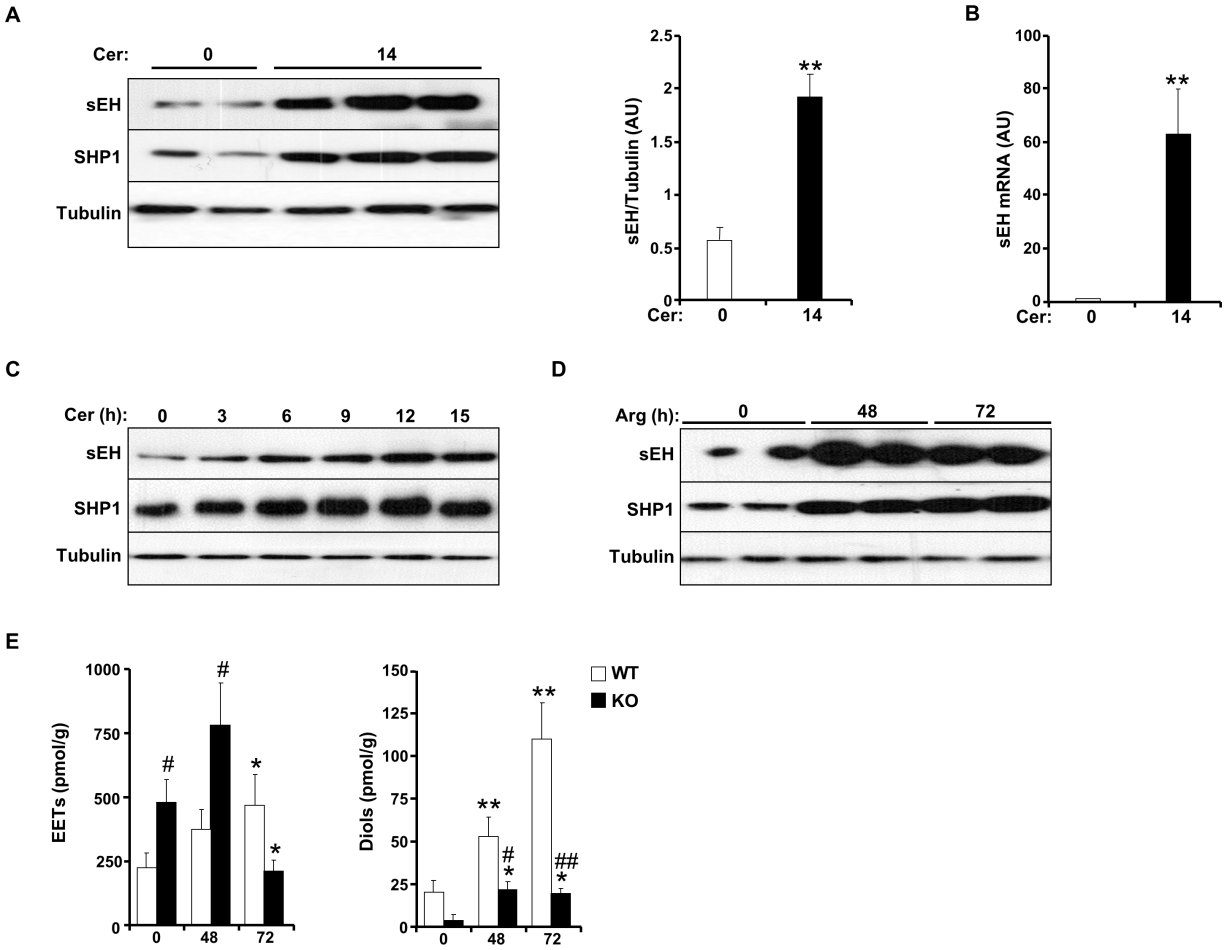
Increased sEH expression in acute pancreatitis. **A**) Total pancreas lysates of wild type mice without and with cerulein administration (14 h) immunoblotted for sEH, SHP1 and Tubulin as a loading control. Representative immunoblots are shown. Bar graph represents expression of sEH (normalized to Tubulin) and presented as means ± SEM (n = 12 per group) (AU: arbitrary units). **B**) *sEH* expression as assessed by quantitative real time PCR in pancreata of wild type mice without (n = 6) and with (n = 6) cerulein. For **A** and **B**, (**: P≤0.01) indicates significant difference between mice without and with cerulein administration. **C**) Total pancreas lysates of wild type mice without and with cerulein administration for the indicated times immunoblotted for sEH, SHP1 and Tubulin. Representative immunoblots are shown. **D**) Total pancreas lysates of wild type mice without and with arginine administration for the indicated times immunoblotted for sEH, SHP1 and Tubulin. **E**) Levels of EET and Diol in wild type and *Ephx2* KO mice without (0) and with (48 and 72 h) arginine administration (n = 4 mice per group). (*: P≤0.05; **: P≤0.01) indicate significant difference between mice without and with arginine administration, and (#: P≤0.05; ##: P≤0.01) indicate significant difference between WT and KO mice.

Expression of pancreatic sEH was determined in wild type mice without and with cerulein-induced pancreatitis. AP was induced in mice with repetitive intraperitoneal injections of cerulein as detailed in Methods. Immunoblots of pancreatic lysates revealed significant increase in sEH expression upon cerulein administration (Fig. 1A). As control, expression of the SH2 domain-containing phosphatase SHP1 was determined since it is increased after cerulein administration [37], [38]. Indeed, pancreatic SHP1 expression increased in mice with cerulein administration (Fig. 1A). In addition, mRNA of the gene encoding sEH, as determined by real time RT-PCR, was increased in the pancreas upon cerulein administration (Fig. 1B). To evaluate the dynamic regulation of pancreatic sEH expression, sEH protein was determined at 3, 6, 9, 12 and 15 h after the initial injection of cerulein. sEH expression increased by 3 h of cerulein administration with progressive increase at later times (Fig. 1C). To ensure that these observations were not limited to a particular model of AP, pancreatic sEH protein expression was also determined in arginine-induced AP model. Similarly, pancreatic sEH expression significantly increased at 48 and 72 h after arginine injection (Fig. 1D). Further, to determine whether the observed increase in sEH expression is mirrored by an increase in enzyme activity, levels of EETs and DHETs were evaluated in pancreata of arginine-treated and untreated mice as detailed in Methods. As expected, KO mice exhibited elevated levels of EETs and decreased levels of DHETs (Fig. 1E). In addition, and consistent with elevated sEH expression during AP, levels of DHETs progressively increased with arginine administration in control mice. Together, these findings reveal increased sEH expression in two rodent models of AP and this was associated with increased sEH activity.

### sEH deficiency mitigates cerulein-induced acute pancreatitis

**Figure 2:**
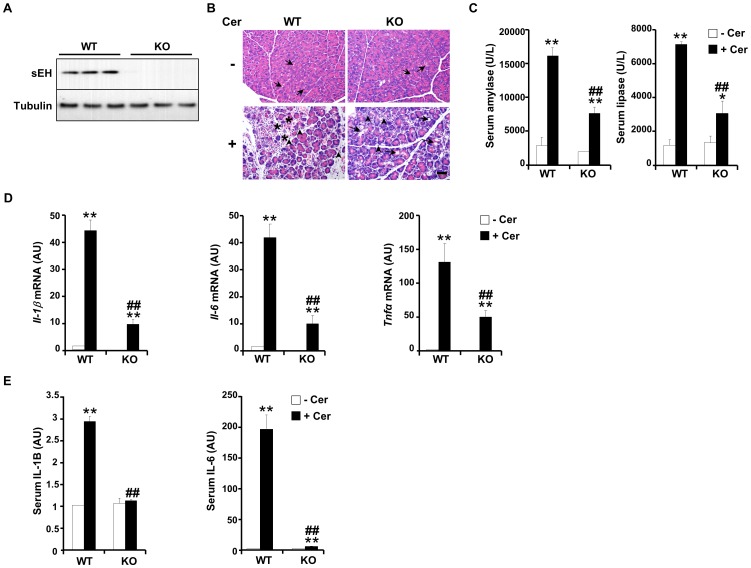
sEH deficiency decreases cerulein-induced pancreatic injury. **A**) Total pancreas lysates from wild type (WT) and *Ephx2* KO mice were immunoblotted for sEH and Tubulin as a loading control. **B**) Acute pancreatitis was induced by intraperitoneal injections of cerulein as detailed in Methods. Representative hematoxylin and eosin (H&E)-stained sections of the pancreas. Upper left: Non cerulein treated WT control – Focal area of acini with intracyoplasmic vacuolation (arrows;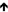). Upper right: Non cerulein treated KO control – Similar to WT control, focal area of acini with intracytoplasmic vacuolation (arrows;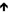). Lower left: Cerulein treated WT mouse 48 h after initial cerulein injection - Lobules and acini are separated by clear spaces (edema) and inflammatory cells (predominantly neutrophils) (arrowheads;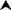), and scattered exocrine cells are necrotic (stars;*). Lower right: Cerulein treated *Ephx2* KO mouse 48 h after initial cerulein injection. Lobules are separated by clear spaces (edema) and contain inflammatory cells (predominantly neutrophils) (arrowheads;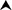). Scattered acinar cells in several lobules have intracytoplasmic vacuolation (arrows;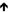). Scale bar: 50 µm. **C**) Serum amylase and lipase were determined in WT mice without (n = 6) and with (n = 6) cerulein and in KO mice without (n = 6) and with (n = 6) cerulein from two independent experiments. **D**) *Il-1b*, *Il-6* and *Tnfa* (as assessed by quantitative real time PCR) in the pancreata of WT mice without (n = 6) and with (n = 6) cerulein and KO mice without (n = 6) and with (n = 6) cerulein. Data are expressed as fold change relative to control (WT without cerulein). **E**) Circulating levels of IL-1B and IL-6 in serum of WT mice without (n = 4) and with (n = 4) cerulein and KO mice without (n = 4) and with (n = 4) cerulein. Data are expressed as fold change relative to control (WT without cerulein). (*: P≤0.05; **: P≤0.01) indicate significant difference between mice without and with cerulein administration, and (##: P≤0.01) indicates significant difference between WT and KO mice.

Increased sEH expression in the early phase of pancreatitis prompted us to determine the role of this enzyme in AP. To that end, we utilized *Ephx2* whole-body KO mice. Immunoblot analysis of total pancreas lysates demonstrated ablation of sEH expression in KO mice compared with controls (Fig. 2A). Thus, this model provides a useful platform to investigate the potential contribution of sEH to AP. To determine the role of sEH during AP, we assessed the severity of cerulein-induced pancreatitis in control and *Ephx2* KO mice as described in Methods. Histological analysis was performed on H&E-stained pancreata sections from WT and *Ephx2* KO mice with and without cerulein administration to evaluate pathological changes including edema, cell vacuolation, inflammation and necrosis (Fig. 2B and Table 2). In most cases, changes when present were localized to the periphery of the pancreatic lobes. As expected, in WT mice cerulein administration caused a significant increase in edema, vacuolation, inflammation and necrosis (Table 2). On the other hand, *Ephx2* KO mice exhibited a significant decrease in cerulein-induced edema, vacuolation and necrosis compared with WT mice (Table 2). In line with the histological analysis, serum amylase and lipase that are markers for AP were significantly different between control and *Ephx2* KO mice. Under basal conditions, serum amylase and lipase were comparable between control and KO mice (Fig. 2C). Cerulein administration led to significant increase in amylase and lipase; however sEH deficiency significantly reduced cerulein-induced serum amylase and lipase. It is worth noting that comparable findings were observed in an independent cohort of mice (data not shown). During AP activation of NF-κB enhances the release of pro-inflammatory cytokines such as IL-1B and IL-6 and TNFA. Accordingly, pancreatic mRNA levels of *Il-1b*, *Il-6* and *Tnfa* were increased in control mice after cerulein administration and this was significantly reduced in KO mice (Fig. 2D). Similarly, serum levels of IL-1B and IL-6 were increased in control mice after cerulein administration and were significantly reduced in KO mice (Fig. 2E). Collectively, these data demonstrate that sEH deficiency mitigates cerulein-induced AP in mice.

**Table 2:** Histological scoring of pancreatic tissues.

		Edema	Vacuolation	Inflammation	Necrosis
**WT**	Ctr n = 14	0.28±0.22	0.5±0.17	0.35±0.22	0.07±0.07
	Cer n = 15	2.26±0.22**	1.46±0.21*	1.00±0.23*	0.53±0.16**
**KO**	Ctrn = 12	0.00±0.00	0.33±0.14	0.00±0.00	0.00±0.00
	Cern = 12	1.25±0.39**^#^	0.50±0.19^##^	0.83±0.29*	0.00±0.00^##^

Hematoxylin and Eosin stained pancreas sections were observed and scored to grade the extent of acinar edema, cell vacuolation, inflammation and acinar cell necrosis. Data are presented as means±SEM. (*: P≤0.05, **: P≤0.01) indicate significant difference between mice without and with cerulein administration (48 h after initial injection), and (#: P≤0.05; ##: P≤0.01) indicate significant difference between WT and *Ephx2* KO male mice.

### sEH deficiency decreases cerulein- and arginine-induced NF-κB inflammatory response

**Figure 3:**
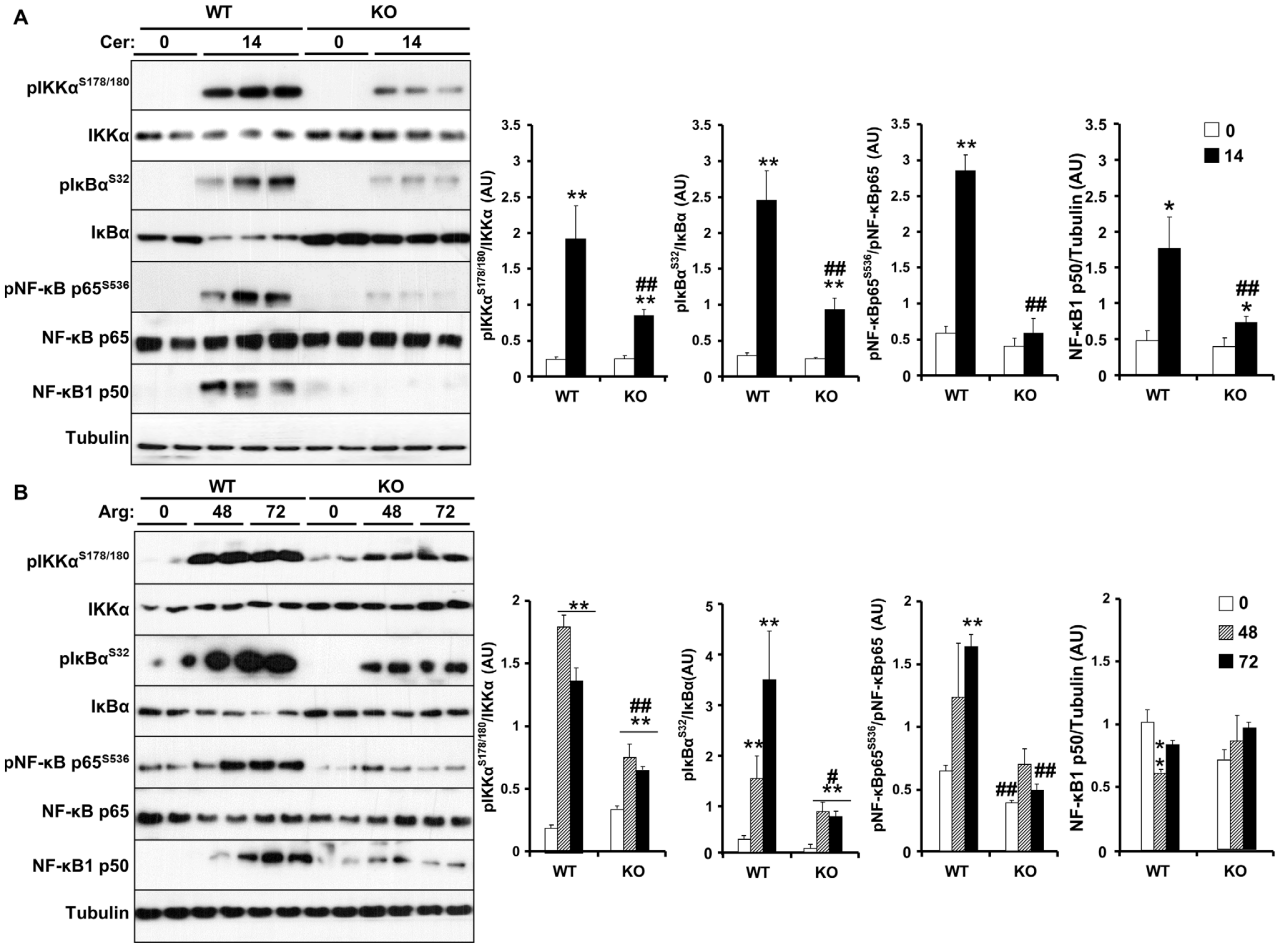
Regulation of cerulein- and arginine-induced NF-κB inflammatory response by sEH. **A**) Total pancreas lysates from wild type mice without (n = 6) and with (n = 9) cerulein, and *Ephx2* KO mice without (n = 6) and with (n = 9) cerulein were immunoblotted for pIKKα, pIκBα, pNF-κB and their respective unphosphorylated proteins, NF-κBp50 and Tubulin as a loading control. Representative immunoblots (n = 2–3 samples per group) are shown. Bar graphs represent normalized data for pIKKα/IKKα, pIκBα/IκBα, pNF-κB/NF-κB and NF-κBp50/Tubulin as means±SEM (AU: arbitrary units). (*: P≤0.05; **: P≤0.01) indicate significant difference between mice without and with cerulein administration, and (##: P≤0.01) indicates significant difference between WT and KO mice. **B**) Total pancreas lysates from wild type mice without (n = 8) and with (n = 8) arginine administration for the indicated times, and *Ephx2* KO mice without (n = 8) and with (n = 8) arginine administration were immunoblotted for pIKKα, pIκBα, pNF-κB and their respective unphosphorylated proteins, NF-κBp50 and Tubulin. Representative immunoblots (n = 2–3 samples per group) are shown. Bar graphs represent normalized data for pIKKα/IKKα, pIκBα/IκBα, pNF-κB/NF-κB and NF-κBp50/Tubulin as means±SEM (AU: arbitrary units). (**: P≤0.01) indicates significant difference between mice without and with arginine administration, and (#: P≤0.05; ##: P≤0.01) indicates significant difference between WT and KO mice.

To investigate the molecular basis for decreased AP in *Ephx2* KO mice, we initially determined alterations in NF-κB signaling. NF-κB is activated early in AP in leukocytes and acinar cells and plays an important role in disease pathogenesis [39]–[41]. sEH deficiency or pharmacological inhibition stabilizes EETs and other fatty acid epoxides which have anti-inflammatory effects through inhibition of NF-κB [28], [29]. Accordingly, we determined the activation status of components of NF-κB signaling pathway in control and KO mice. Cerulein-induced IKKα, I_k_Bα and NF-κBp65 phosphorylation and NF-κBp50 expression were attenuated in *Ephx2* KO mice compared with controls (Fig. 3A). Similarly, arginine-induced IKKα, I_κ_Bα and NF-κBp65 phosphorylation and NF-κBp50 expression were attenuated in KO mice compared with controls (Fig. 3B). These data demonstrate decreased cerulein- and arginine-induced NF-κB inflammatory response in mice with sEH deficiency. This is in keeping with the reduced pancreatic and circulating pro-inflammatory cytokines in cerulein-treated KO mice.

### sEH deficiency decreases cerulein- and arginine-induced MAPKs signaling and cell death

**Figure 4:**
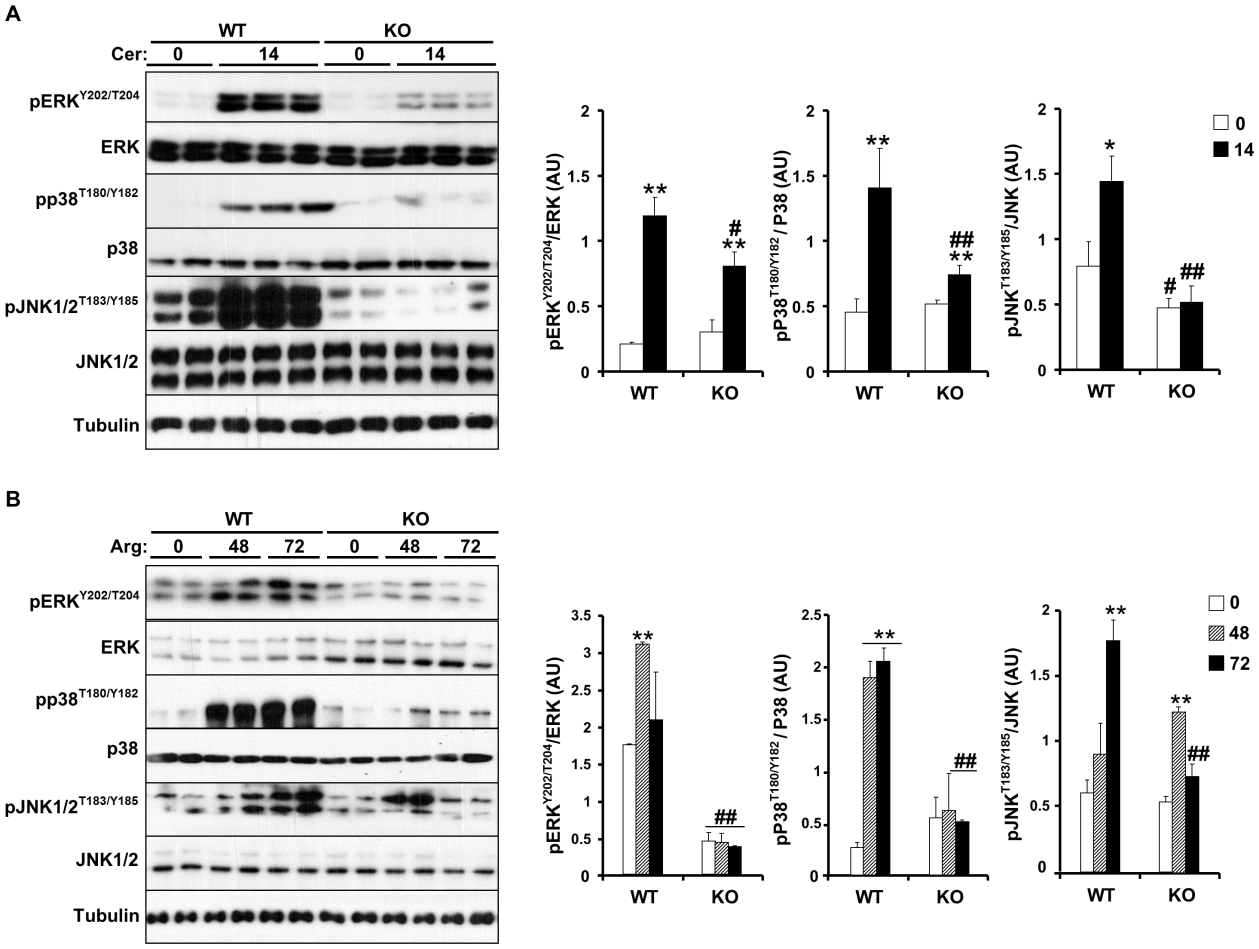
sEH deficiency decreases cerulein- and arginine-induced MAPKs signaling. **A**) Total pancreas lysates from wild type mice without (n = 6) and with (n = 9) cerulein, and *Ephx2* KO mice without (n = 6) and with (n = 9) cerulein were immunoblotted for pERK1/2, pp38, pJNK1/2 and their respective unphosphorylated proteins and Tubulin as a loading control. Representative immunoblots (n = 2–3 samples per group) are shown. Bar graphs represent normalized data for pERK/ERK, pp38/p38, and pJNK/JNK, and presented as means±SEM (AU: arbitrary units). (*: P≤0.05; **: P≤0.01) indicate significant difference between mice without and with cerulein administration, and (#: P≤0.05; ##: P≤0.01) indicate significant difference between WT and KO mice. **B**) Total pancreas lysates from wild type mice without (n = 8) and with (n = 8) arginine administration for the indicated times, and *Ephx2* KO mice without (n = 8) and with (n = 8) arginine administration were immunoblotted for pERK1/2, pp38, pJNK1/2 and their respective unphosphorylated proteins and Tubulin. Representative immunoblots (n = 2–3 samples per group) are shown. Bar graphs represent normalized data for pERK/ERK, pp38/p38, and pJNK/JNK, and presented as means±SEM. (**: P≤0.01) indicate significant difference between mice without and with arginine administration, and (##: P≤0.01) indicates significant difference between WT and KO mice.

**Figure 5:**
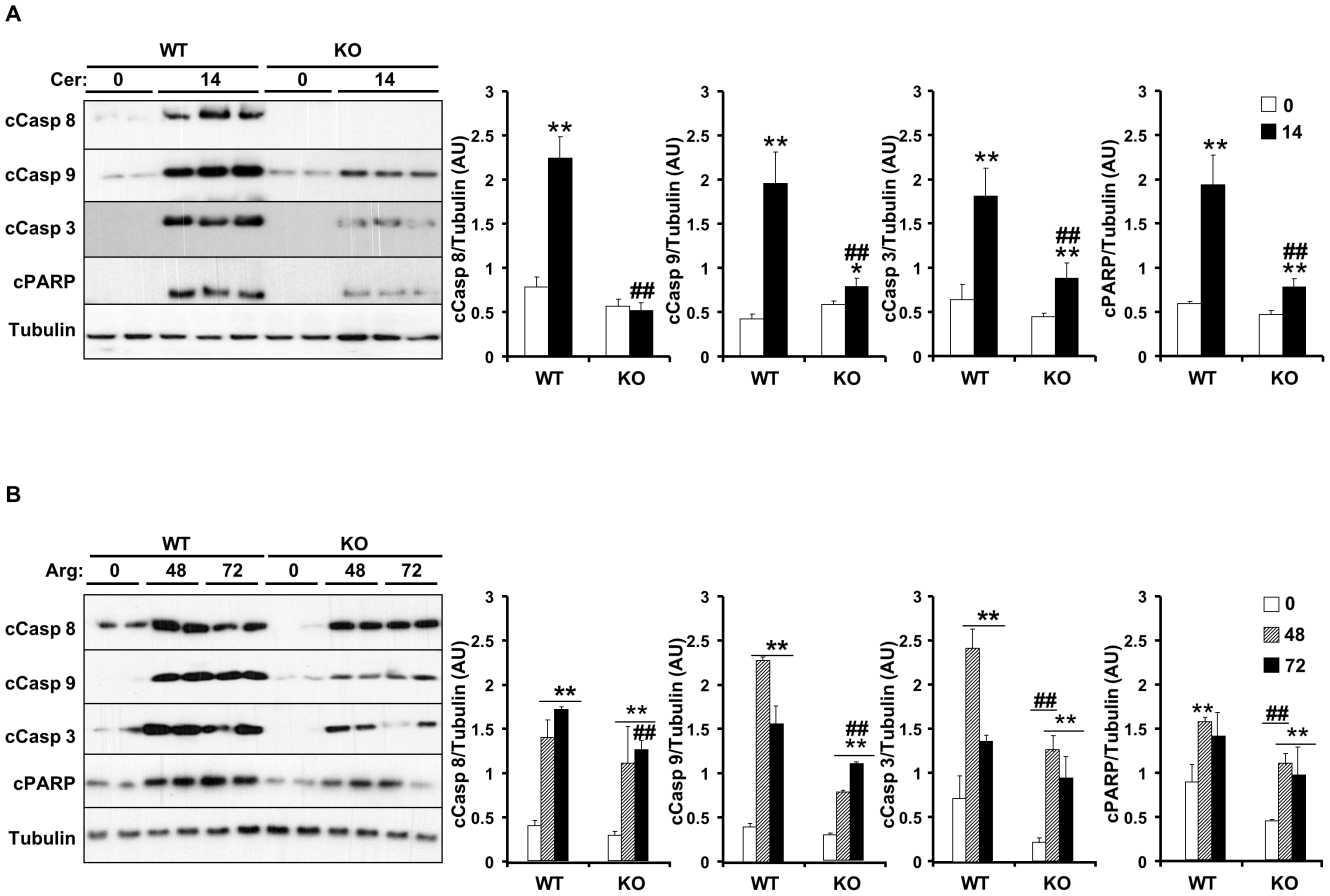
sEH deficiency attenuates markers of cerulein- and arginine-induced cell death. **A**) Total pancreas lysates from wild type mice without (n = 6) and with (n = 9) cerulein, and *Ephx2* KO mice without (n = 6) and with (n = 9) cerulein were immunoblotted for cleaved Caspases 8, 9 and 3, PARP and Tubulin as a loading control. Representative immunoblots (n = 2–3 samples per group) are shown. Bar graphs represent normalized data for Caspases 8, 9, 3 and PARP/Tubulin as means±SEM (AU: arbitrary units). (*: P≤0.05; **: P≤0.01) indicate significant difference between mice without and with cerulein administration, and (##: P≤0.01) indicates significant difference between WT and KO mice. **B**) Total pancreas lysates from wild type mice without (n = 8) and with (n = 8) arginine administration for the indicated times, and *Ephx2* KO mice without (n = 8) and with (n = 8) arginine administration were immunoblotted for Caspases 8, 9 and 3, PARP and Tubulin. Representative immunoblots (n = 2–3 samples per group) are shown. Bar graphs represent normalized data for Caspase 8, 9, 3 and PARP/Tubulin as means±SEM. (**: P≤0.01) indicate significant difference between mice without and with arginine administration, and (##: P≤0.01) indicates significant difference between WT and KO mice.

Mitogen-activated protein kinases (MAPKs) including p38, ERK1/2 and JNK1/2 are induced rapidly and transiently during experimental AP in rodents [42]. This activation is believed to be a component of the cellular stress response in the onset of inflammation in the pancreas. Treatment with EETs reduces inflammation-induced p38 phosphorylation to mediate anti-inflammatory properties [43]. Cerulein administration led to increased phosphorylation of ERK, p38 and JNK in control mice and that was significantly decreased in *Ephx2* KO mice (Fig. 4A). Similarly, arginine administration increased ERK, p38 and JNK phosphorylation in control mice and that was significantly decreased in *Ephx2* KO mice (Fig. 4B). After exposure to apoptotic stimuli, cells activate initiator Caspases (Caspases 8 and 9) that proteolytically cleave and activate effector Caspases (Caspases 3 and 7) to dismantle dying cells [44], [45]. Accordingly, we assessed cerulein-induced expression of initiator and effector Caspases in control versus *Ephx2* KO mice. Cerulein caused pro-Caspases 8, 9 and 3 cleavage and an increase in the cleavage fragments and induced cleavage of Caspase 3 substrate; poly (ADP-ribose) polymerase (PARP) (Fig. 5A). sEH deficiency decreased cleaved Caspase 8, 9 and 3 and PARP expression indicative of decreased apoptosis (Fig. 5A). In addition, comparable findings were observed in arginine-treated cohort (Fig. 5B). Collectively, these findings demonstrate decreased MAPKs signaling and cell death upon sEH deficiency during the early phase of cerulein- and arginine-induced AP.

## Discussion

The development of AP involves a complex cascade of events that are triggered by acinar cells, but the underlying mechanisms regulating the initiation and severity of the disease are not well understood. In the current study, we investigated the role of sEH in AP using two rodent models cerulein- and arginine-induced AP. We report increased sEH expression and activity during the early phase of AP. Importantly, sEH deficiency mitigated the effects of cerulein- and arginine-induced AP in mice. This was associated with decreased cerulein- and arginine-induced NF-κB inflammatory response and decreased cell death in *Ephx2* KO mice. Together, these findings demonstrate a novel role for sEH in the pancreas and suggest that sEH pharmacological inhibition may be of therapeutic value in AP.

Alterations in gene and protein expression during the initiation phase of AP play a significant role in the progression and severity of the disease [46]. In this regard, we observed increased sEH mRNA and protein expression in a cerulein-induced AP mouse model. This model was utilized since secretagouge-induced pancreatitis, generated by administration of supramaximally stimulating dose of cerulein, is very well characterized and has characteristics that are similar to those of human pancreatitis [7]. Of note, these findings were recapitulated in arginine-induced AP indicating that they were not unique to a particular rodent model of AP. Moreover, increased sEH expression during AP was mirrored by comparable changes in enzyme activity. While additional studies are required to establish if sEH expression and activity are comparably regulated in human AP, it is worth noting that increased hepatic and adipose sEH expression in HFD-fed mice was mirrored by increased sEH expression in overweight humans [47].

Using a genetic approach, we demonstrated that sEH deficiency ameliorated the course of AP as evidenced by pancreas histology, reduced amylase and lipase, decreased pancreatic *Il-1b*, *Il-6* and *Tnfa* expression and decreased serum levels of IL-1B, IL-6. Pro-inflammatory cytokines play a pivotal role in the progression and severity of pancreatitis [12], [13], [48]. TNFA exacerbates acinar cell injury, IL-1B plays a role in the development of AP and IL-6 is a major mediator of the acute-phase response. Further, suppression of these pro-inflammatory cytokines could attenuate the severity of pancreatitis [49]. It remains unclear if the decreased expression of such pro-inflammatory cytokines in *Ephx2* KO mice may be associated with alterations in expression of anti-inflammatory cytokines. Nevertheless, it is reasonable to stipulate that the protective effects of sEH deficiency could be mediated, at least in part, through the attenuation of the inflammatory response. It is important to note that since *Ephx2* KO mice exhibit global sEH deficiency the inflammatory response is likely regulated by the systemic effects of sEH deletion. Accordingly, additional studies are warranted to determine the effects of specific pancreatic sEH deficiency on cytokine expression and development of AP.

sEH deficiency modulated cerulein- and arginine-induced NF-κB inflammatory response and MAPKs signaling. NF-κB inflammatory response is activated early in AP and plays an important role in disease pathogenesis [39]–[41]. In addition, sEH deletion correlated with decreased activation of the MAPKs ERK1/2, p38 and JNK indicative of decreased stress and is in line with previous studies implicating MAPKs in AP [50]–[53]. The precise mechanism by which sEH deficiency attenuates MAPK signaling remains unclear, but can be indirect and related to reduced inflammation. sEH deficiency may impact on additional signaling pathways that have been previously implicated in pancreatitis. For example, endoplasmic reticulum (ER) stress has been implicated in the pathophysiology of pancreatitis, in particular alcohol-induced pancreatic damage [54]. Previously, we reported attenuation of HFD-induced ER stress in adipose and liver upon sEH deficiency [47]. Thus, the effects of sEH deficiency or pharmacological inhibition on ER stress during AP warrant additional investigation.

The current studies suggest that sEH inhibition in the pancreas may represent a potential approach for treating acute pancreatitis; however it is important to note that the effects of pancreas-specific sEH deficiency on AP remain to be determined. Further, the therapeutic effects of sEH pharmacological inhibition after the development of AP need to be evaluated. Nevertheless, the findings presented herein uncover a novel role for sEH in AP and suggest that interventions designed to inhibit pancreatic sEH may be of value in combating this disease.
